# SeQual-Stream: approaching stream processing to quality control of NGS datasets

**DOI:** 10.1186/s12859-023-05530-7

**Published:** 2023-10-27

**Authors:** Óscar Castellanos-Rodríguez, Roberto R. Expósito, Juan Touriño

**Affiliations:** Universidade da Coruña, CITIC, Computer Architecture Group, Campus de Elviña, 15071 A Coruña, Spain

**Keywords:** Quality control, Big data, Stream processing, Apache Spark, Next generation sequencing (NGS)

## Abstract

**Background:**

Quality control of DNA sequences is an important data preprocessing step in many genomic analyses. However, all existing parallel tools for this purpose are based on a batch processing model, needing to have the complete genetic dataset before processing can even begin. This limitation clearly hinders quality control performance in those scenarios where the dataset must be downloaded from a remote repository and/or copied to a distributed file system for its parallel processing.

**Results:**

In this paper we present SeQual-Stream, a streaming tool that allows performing multiple quality control operations on genomic datasets in a fast, distributed and scalable way. To do so, our approach relies on the Apache Spark framework and the Hadoop Distributed File System (HDFS) to fully exploit the stream paradigm and accelerate the preprocessing of large datasets as they are being downloaded and/or copied to HDFS. The experimental results have shown significant improvements in the execution times of SeQual-Stream when compared to a batch processing tool with similar quality control features, providing a maximum speedup of 2.7$\times$ when processing a dataset with more than 250 million DNA sequences, while also demonstrating good scalability features.

**Conclusion:**

Our solution provides a more scalable and higher performance way to carry out quality control of large genomic datasets by taking advantage of stream processing features. The tool is distributed as free open-source software released under the GNU AGPLv3 license and is publicly available to download at https://github.com/UDC-GAC/SeQual-Stream.

## Background

Obtaining DNA sequences from living beings is usually the first step in the studies developed by biologists and bioinformaticians. The continuous development of Next Generation Sequencing (NGS) technologies [1] during the last decade has led to a vertiginous increase in the amount of available genomic data. Hundreds of millions of sequences (the so-called reads) can now be generated in a single experiment at a drastically reduced cost. However, the accuracy of current NGS platforms is not high in all cases. The quality of downstream analyses may be affected because of the artifacts introduced in some DNA fragments during the sequencing process [2, 3], regardless of the NGS platform. Therefore, quality control is an essential preprocessing step for raw NGS data [4], removing or modifying those input reads that are not considered useful.

In this paper we introduce SeQual-Stream, a parallel tool implemented in Java that allows performing multiple quality control operations (e.g., trimming, filtering) on large genomic datasets in a distributed and scalable way. To do so, it takes full advantage of the Apache Spark Big Data framework [5] together with the Hadoop Distributed File System (HDFS) [6]. Up to our knowledge, all existing parallel quality control tools operate on a batch processing model, which means that they require the entire input dataset before any data processing can begin. This poses a performance constraint, as downloading the data from a remote repository and copying them to a distributed file system such as HDFS for parallel processing are costly operations that significantly delay the start of the quality control. This problem is especially relevant in the NGS context as the size of the genomic datasets is continuously increasing, which demands more efficient processing modes. To overcome this issue, SeQual-Stream has been implemented upon the Spark Structured Streaming API [7], in order to apply the quality control operations to the input reads as the data are being downloaded from a remote location (e.g., a web repository) and/or copied to HDFS. This stream-based processing mode significantly reduces runtimes by enabling efficient overlapping of download and copy operations to HDFS with the actual data processing performed by SeQual-Stream.

The main contributions of this paper over the state of the art are the following:

- Up to our knowledge, we introduce the first quality control tool that can exploit the stream processing model to accelerate the preprocessing of raw NGS datasets.
- We conduct an extensive experimental evaluation on two cluster testbeds using publicly available real-world datasets, both to demonstrate the performance benefits of our approach compared to a batch processing quality control tool and to analyze its scalability.
- SeQual-Stream is implemented in “pure” (100%) Java code in order to maximize cross-platform portability, whereas supporting standard unaligned sequence formats (FASTQ/FASTA). The tool is publicly available under a GNU AGPLv3 license.

### Quality control

Current NGS technologies can generate a huge number of DNA segments massively and in parallel, in less time and at a lower cost per base than previous platforms. However, they also introduce errors in some sequences, making them not useful for downstream analyses. So, quality control consists of removing those sequences or modifying them to be useful in order to improve subsequent processing (e.g., sequence alignment). Multiple operations can be performed within the context of quality control. For example, different filtering criteria can be applied when removing sequences according to their length (i.e., number of bases), the quality scores of the bases, the proportion of content of certain bases, or according to the occurrence of certain base patterns. When modifying sequences, trimming techniques can be used so that they are trimmed, for instance, to a certain average quality or to a maximum length. Another example is formatting techniques, such as the conversion from FASTQ to FASTA or between DNA and RNA, as well as renaming the sequence identifiers, among others.

### Big data

The great development of NGS has led to a significant increase in the amount of genomic data to be processed, making the concept of Big Data a fundamental asset in current biomedicine. Big Data often refers to a massive volume of both structured and unstructured data that is so large and difficult to process using traditional methods and systems. The rise of Big Data is usually associated to novel technologies and algorithms such as MapReduce [8], a parallel programming paradigm characterized by being divided into two distinct phases: Map and Reduce. These sub-processes are executed in a distributed manner relying on a cluster of nodes. MapReduce is also the name of the Google’s proprietary implementation, who first proposed the model in 2004, given the need to optimize user search results on the web. To support this type of processing, a distributed data storage system, based on storing data on more than one node, is used. Google proposed the Google File System (GFS) [9], a high-performance distributed file system that follows a master/worker architecture. Following this model, other technologies implementing it came to light. Of particular note is the open-source Apache Hadoop framework [10]. Hadoop integrates HDFS [6] as storage layer to distribute files across the cluster divided into data blocks, thus providing the prior division of the data needed by the Hadoop MapReduce data processing engine, in addition to replicating those blocks to provide fault tolerance. Both MapReduce and Hadoop are intended for batch processing, since they require the input data to be stored completely in a distributed manner before processing begins.

#### Apache Spark and stream processing

Apache Spark [5] is an open-source, general-purpose framework for distributed processing designed to be simple and fast. Spark emerges as an evolution of Hadoop, which is very limited in terms of processing modes and performance. Some of the improvements that Spark brings over its predecessor are in-memory computing, support for stream processing and the ease to interact with multiple persistent storage systems, such as HDFS or Cassandra [11].

Spark provides a fundamental data abstraction for distributed processing: the Resilient Distributed Dataset (RDD) [12]. An RDD is defined as a collection of elements partitioned across the cluster nodes and capable of operating in parallel. Two alternatives to RDDs are currently offered by Spark: DataFrames and Datasets [13]. DataFrames organize data in columns, similar to a table in a relational database. Unlike RDDs, they allow better handling of structured data and are able to optimize the queries performed. Datasets are an extension of DataFrames and try to combine the advantages of RDDs and DataFrames in the same API, i.e., the ease of use of RDDs and the performance optimization of DataFrames.

The key Spark feature for the development of SeQual-Stream is the support for stream processing by providing the Structured Streaming API [7], as opposed to its batch API. Structured Streaming was designed as an evolution of the legacy Spark Streaming API [14]. This legacy API represents the continuous data stream with a high-level abstraction called discretized stream (DStream), which is represented internally as a sequence of RDDs. Spark Streaming follows a micro-batch model, consisting of polling the source and dividing the input data into small batches that are processed using the batch API, thus generating batches of processed data. However, Structured Streaming brings several improvements over its predecessor. This API is built on top of the Spark SQL library, using higher-level DataFrames and Datasets as data abstractions. It is also capable of correctly processing data that arrive late to the processing engine. Legacy Spark Streaming only takes into account the timestamp of data reception by Spark, so if an event “a” arrives later than a subsequent event “b”, we would be losing accuracy in the information, which can be equal to data loss. However, Structured Streaming is able to process out-of-order data correctly if it includes a timestamp, thus generating consistent results.

**Fig. 1:**
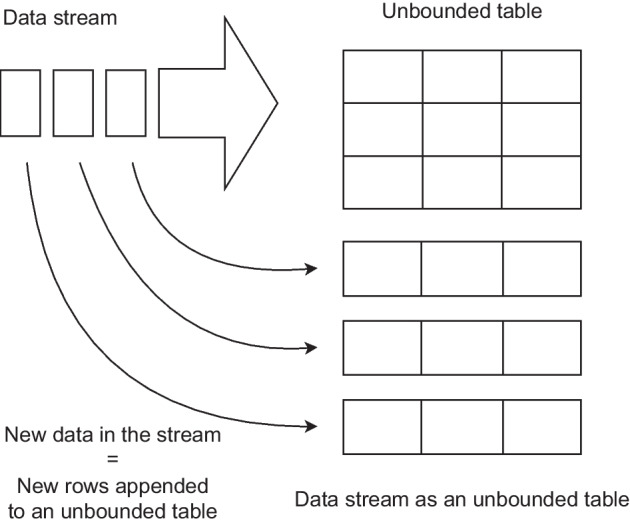
Structured streaming model in Apache Spark

By default, Structured Streaming queries are also processed internally using a micro-batch model, with the important difference that it treats the input data stream as an unbounded table that is continuously being appended by new rows, as shown graphically in Fig. 1. The API also allows to choose an alternative processing mode called Continuous Processing, which can achieve lower end-to-end latencies, although it is still in an experimental state. Its operation is based on constantly reading the source and processing the data as soon as they are available, instead of polling the source periodically. Another feature is allowing stream jobs to be expressed in the same way as a batch job with static data. The Spark SQL engine takes care of executing it incrementally and continuously, updating the final result as the data arrive. Each time the result table is updated, it is written (persisted) to an external system, which can be a file or a Kafka topic [15], among other options. Depending on the data to be written to the output, three modes are differentiated:

- Complete mode: all table rows are written in each update.
- Append mode: only new rows are written. This makes it only applicable when existing rows are expected to remain unchanged. This is the default mode.
- Update mode: the rows that have changed since the last table update are written.

## Related work

There is a wide variety of tools within the context of bioinformatics. Many of them follow a batch processing model, such as CloudEC [16] for error correction or BigBWA [17] for sequence alignment, both of them Big Data tools relying on the Apache Hadoop framework.

Focusing on tools for performing quality control, all existing approaches are based on batch processing. Examples of such tools are FASTX-Toolkit [18], PRINSEQ [19], LongQC [20] and iSeqQC [21], which do not provide any support for parallel processing. QC-Chain [22] and PRINSEQ++ [23] does provide such parallel support through multithreading, and so their scalability is limited to a single node, whereas FastQC [24] and Falco [25], which is an emulation of the former, only support parallelism at the file level. SOAPnuke [26] is able to distribute the data processing to a cluster of nodes through Hadoop, whereas SeQual [27] is also capable of scaling out across a cluster by relying on the more efficient Spark RDDs, greatly enhancing performance compared to previous solutions. Nevertheless, both SOAPnuke and SeQual are still limited by the batch processing operation mode they are based on. In terms of functionality, we have taken SeQual as reference for developing SeQual-Stream due to its wide range of supported quality control operations (more than 30), one of the largest among the state of the art.

It is also interesting to mention other bioinformatics tools that follow a stream processing model for other purposes. For instance, we can find in the literature several solutions for estimating the number of k-mers in genomic datasets, such as KmerStream [28], ntCard [29], KmerEstimate [30] and Khmer [31]. Other tools are focused on sequence alignment (StreamAligner [32], StreamBWA [33]), metagenomics profiling (Flint [34]) and DNA analysis (SparkGA2 [35]). These latter examples are all implemented on top of the legacy Spark Streaming API instead of using Spark Structured Streaming as in our approach.

Finally, as our tool is based on Spark, we consider it important to highlight the usefulness of this Big Data framework in real biological studies. For example, SparkGATK [36], a framework for DNA analysis, has been used to detect allele-specific expression genes in plants [37]. SparkBWA [38], a DNA sequence alignment tool, has been used in [39] as part of the analysis pipeline to construct a high-density genetic linkage map for the common bean and identify a gene resistant to the bruchid, as well as to analyze the growth plasticity of bacteria in [40].

## Implementation

SeQual-Stream is a parallel Java tool that provides a wide set of operations to apply quality control and data preprocessing on raw NGS data. These operations are grouped into three different categories depending on the functionality they provide: (1) single filters, responsible for discarding input sequences that do not meet a certain criteria (e.g., sequence length), evaluating each sequence independently of the others; (2) trimmers, operations that trim certain sequence bases at the beginning or end; and (3) formatters, operations to change the format of the input dataset (e.g., from DNA to RNA). The tool can receive as input single- or paired-end datasets, supporting FASTQ and FASTA formats. The input files can be stored either in HDFS or locally. It is important to note that there is no real dependency on HDFS as such, but on the Hadoop API, which is fully integrated into Spark. So our tool supports any other file system that is compatible with such an API. In fact, the local file system is just one of the available implementations in addition to HDFS or Amazon S3. For simplicity, we will keep using the term “HDFS” from now on, as it is also the file system used in the experimental evaluation. The datasets may be complete or in the process of being downloaded from a remote server, since SeQual-Stream can process data as new sequences continue to arrive. Note that in the case of having the complete files stored locally, they are also processed in a streaming way as they are being copied to HDFS. Furthermore, our tool features a graphical user interface to provide greater convenience to bioinformaticians and biologists (see Additional file 1: Fig. S1 ).

**Fig. 2:**
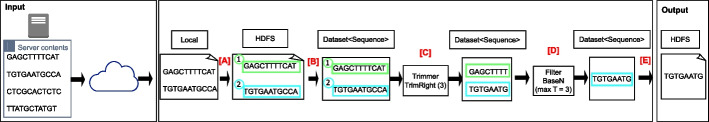
Overview of the SeQual-Stream dataflow

Figure 2 shows an overview of the SeQual-Stream dataflow to perform two quality control operations: a trimmer and a filter. In this example, there are only four DNA sequences stored in a remote server and we only show their bases for simplicity. At a certain moment, two of them have already been downloaded in a local file, so that SeQual-Stream can begin to process them by copying the available sequences to HDFS (labeled as “[A]” in the figure). Next, a Spark Dataset containing those sequences is created (“[B]”) to be operated by the trimmer and the filter (“[C]” and “[D]”, respectively). In this example, the trimmer operation is TrimRight, which trims a certain number of bases from each sequence starting from the right (three bases in this case). The filter operation is BaseN, which filters sequences according to whether or not they contain a maximum and/or minimum number of one or several base types (a maximum of three “T” bases is allowed in this case), discarding the first sequence and leaving at the end a single trimmed sequence in the Spark Dataset. Finally, the resulting sequences are written back to HDFS (“[E]”). When more content is obtained from the server, the local input file will be updated and the entire process is repeated with the new sequences. Note that this process is executed in parallel with a previous or subsequent iteration.

Therefore, this dataflow can be divided into three main stages as follows:

1. Reading of the input dataset(s), which may be stored in HDFS or locally and may be in the process of being downloaded.
2. Processing of the available sequences by applying the quality control operations configured by the user.
3. Writing of the results to the output files using the path specified by the user.

The next sections provide more specific details about the implementation of each stage.

### Reading of the input datasets

The objective of the first stage is the creation of a Spark Dataset that represents in a relational table the sequences to be processed in the next stage. Basically, Structured Streaming operates by indicating a directory in HDFS to be monitored and processing the files as they are written to such directory. The main problem is that once the available data of a certain file has been processed, such file is not processed again even if it is updated with new content. So, it cannot be used to process large files that are still in the process of downloading. To overcome this issue, the proposed solution consists of creating a previous stage in charge of reading the input dataset (“[A]” in Fig. 2) and generating new files formed by subsets of the input data (called “subfiles”) that Structured Streaming is able to process. Note that this reading stage works iteratively. For example, if there is a subset of sequences downloaded at a given time from a certain dataset, this stage will perform a first iteration to store those sequences in a new subfile on HDFS so that Structured Streaming can process it, and then it will wait for the remaining sequences to be downloaded. After a few seconds, it will recheck the state of the input dataset and, if new data is found, the procedure is repeated through a second iteration, generating a new subfile with new sequences to be processed.

It is important to remark that only complete sequences are copied to subfiles. If no more data is available at a given time and the last sequence is incomplete, only the complete sequences before the last one (if any) are copied while waiting for new data to arrive. Therefore, the copy operations cannot be done on a line-by-line basis, since it is necessary to evaluate if sequences are complete as they are represented in multiple lines (e.g., at least two for FASTA format). This process is even more complex for paired-end datasets, where there are two input files to be downloaded. In addition to copy only complete sequences, it must be done synchronously in both files because one of them may have more available data than the other as download speeds may differ. The solution to this issue is reading the sequences in pairs (i.e., only if both are complete) and copy them together within the same subfile.

#### Parallel reading

In order to speed up the previously described process, the reading stage is divided and performed in parallel in one of the cluster nodes through multithreading support. The number of parallel threads used is adapted dynamically by the tool, depending on the computational capacity of the node and the amount of data available at that moment in order to avoid the overhead that would be generated by creating a lot of threads that read few data. A limit is imposed on the amount of data to be copied in a single iteration of the reading stage. This is done to improve the overlap of reading the input dataset and processing it with Structured Streaming. Without this limit, if there is a lot of data that have already been downloaded, the cluster nodes will be mostly idle while waiting for the reading stage to copy them all to HDFS.

#### Creation of the Spark dataset

Once new subfiles are copied to HDFS, Structured Streaming is able to automatically detect them to allow SeQual-Stream creating a Spark Dataset of sequences (“[B]” in Fig. 2). Although Spark supports several common file formats (e.g., JSON, CSV), sequence formats cannot be read straightforwardly, and so the standard text-based file format provided by Spark must be used. By default, this format separates the file on a line-by-line basis. However, FASTQ/FASTA sequences are composed of multiple lines, so it would be interesting to use a character that clearly separates each one. The problem is that although FASTQ sequences begin with the “@” character, this character can also appear in the quality scores. To overcome this issue, the reading stage is in charge of adding a specific string before each sequence when copying it into a subfile in order to be used as an unambiguous separator. Once the sequences can be correctly separated, their different parts, such as their identifier and bases, can be obtained unambiguously, and the corresponding Spark Dataset can be created. For paired-end datasets, the separator string must be added to each pair written to the subfile. So, SeQual-Stream is able to differentiate each sequence of the pair when creating the Spark Dataset.

### Processing of the sequences

The next stage of the pipeline is the processing of the sequences contained on a Spark Dataset by applying the quality control operations selected by the user (“[C]” and “[D]” in Fig. 2). As previously mentioned, the functionality supported by our tool is inspired on those operations provided by SeQual [27], but adapted to the stream processing model. The first group of quality control operations consists of 12 single filters that were implemented using the Spark’s *filter* method. Each operation implements the corresponding boolean function to discard those sequences that do not meet a certain criteria. For example, the Length filter evaluates whether the size of the sequence is smaller and/or larger than an upper and/or lower limit configured by the user.

The second and third group of operations (10 trimmers and 3 formatters, respectively) were implemented using the Spark’s *map* method, which allows to process the Dataset by applying a specific function to each element (i.e., sequence). For example, TrimLeft trims a given number of bases from each sequence (and their quality scores if applicable) starting from the left using the Java *substring* method. Another example is DNAToRNA, a formatter that changes the format of each sequence from DNA to RNA by replacing the thymine bases (represented by a “T” character) with uracil (“U” character) using the Java *replace* method.

Note that all the quality control operations are performed as new sequences get loaded into the Spark Dataset, so that the processing stage efficiently overlaps with the reading of the input dataset.

### Writing of the results

After a certain set of quality control operations is performed over the sequences, they must be written back to HDFS (“[E]” in Fig. 2). By default, the output sequences are written throughout different output files. This fact is due to two reasons: (1) when processing the sequences in a Spark Dataset, the processing tasks are executed on different cluster nodes so that each one writes to its corresponding output file (or “part” file); (2) when using our stream processing approach, the sequences are written as soon as the subfiles, which contain subsets of the input files, are processed by SeQual-Stream, and thus each subfile generates multiple part files distributed over the cluster.

**Fig. 3:**
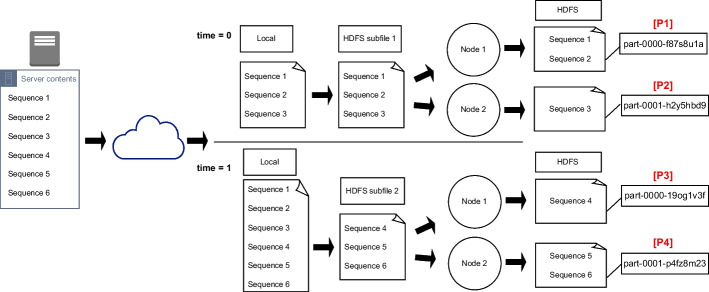
Illustration of the naming order problem with several part files

The main issue of having multiple output files is how to keep the output sequences in the same order that the input. When persisting a Spark Dataset, the different parts are named in alphabetical order so that the original order is maintained. For example, the first part file (“part-0000”) may contain the sequences from 1 to 100, the second one (“part-0001”) from 101 to 200, and so on. The problem arises when using Structured Streaming, since Spark processes each subfile independently and does not preserve any alphabetical naming order between parts generated from different subfiles. Figure 3 illustrates this issue, where two different subfiles are generated at different moments in time from the input dataset being downloaded. In this example, the processing of every subfile is distributed on two nodes, thus generating four part files in total. Parts labeled as “[P1]” and “[P2]” are named in alphabetical order, and the same applies to parts “[P3]” and “[P4]”, but the order is not respected between all of them. A naive solution to this issue would be using the write timestamp of each part file, since the first sequences should be processed and written before the following ones. However, this rule is not consistent: when several subfiles are written to the directory that Structured Streaming is monitoring, there is no guarantee that they will be processed in the same order they were created.

The solution proposed in SeQual-Stream consists of embedding a custom timestamp within each sequence during the reading stage (“[A]” in Fig. 2) so that the order is set from the very beginning. More specifically, SeQual-Stream must be able to differentiate each generated subfile during such reading stage. So, we use a timestamp composed of two integers to indicate, respectively, each iteration of the reading stage and the thread number that generated the corresponding subfile. For example, thread #5 during iteration #3 will embed the timestamp “3–5” in its sequences. Therefore, SeQual-Stream actually processes a Spark Dataset containing sequences tagged with a custom timestamp. Right before writing the results to HDFS, this Spark Dataset is separated into two columns through a *flatmap* operation: the sequences themselves and their timestamps. This approach allows writing the results partitioned by the timestamp column, an operation that consists of gathering the part files containing sequences with the same timestamp into the same output directory. Those parts are already sorted alphabetically, and thus the global order is ensured. For instance, the part files generated from the sequences with timestamp “3–5” are stored in a directory named “timestamp=3–5”. If a single output file containing all the resulting sequences is preferred by the user, our tool allows to configure an option to merge them together in the appropriate order.

Regarding the writing operation itself, it is done through a Spark object called “StreamingQuery” that remains in a loop as long as there is data to be written. This loop ends when the reading stage sends a specific signal meaning that there is no more input data, and when the StreamingQuery has no pending data to write. Finally, the output write mode for Structured Streaming will be “append”, since we are only interested in writing the new processed sequences in a new part file, whereas the already written sequences must not change.

## Results and discussion

The experimental evaluation of our proposal has been conducted on two different testbeds. The first one is a 17-node commodity cluster consisting of one master and 16 worker nodes. The hardware characteristics of each node are shown in Table 1. This cluster provides a total of 256 physical cores and 1 TiB of memory for the processing tasks, and each node has one local hard disk for HDFS and temporary data storage during the execution of the experiments. The second testbed is a more modern, high-performance 9-node cluster consisting of one master and 8 worker nodes. Table 2 shows the characteristics of each node, providing a total of 256 physical cores and 2 TiB of memory for the processing tasks, with each node having two local disks: one large but slow hard disk and one small but fast solid state disk. Tables 1 and 2 also show the software configuration of each testbed, with both systems running CentOS Linux 7.9.2009. As can be observed, the Spark and Hadoop versions used in the experiments were the same in both testbeds. Some specific configuration parameters for Spark and HDFS are also shown in these tables, such as the block size for HDFS and the number of cores for Spark executors, which is adapted to the features of the cluster nodes.

Three publicly available FASTQ datasets have been evaluated, obtained from the Sequence Read Archive (SRA) [41, 42], a public repository of genomic data belonging to the National Center for Biotechnology Information (NCBI) [43, 44]. These datasets present a paired-end layout, so they consist of two input files (single-end experiments use one file). Their main characteristics are summarized in Table 3, where the number of reads (third row) refers to the number of DNA sequences in each input file, and the read length (fourth row) refers to the number of base pairs (bp) per each sequence.

The experimental evaluation has been carried out comparatively with SeQual, the tool used as reference to implement our solution in terms of its functionality, as mentioned before. Four representative quality control operations have been selected for this performance comparison:

- QUALITY: a single filter that filters sequences based on an indicated maximum and/or minimum mean quality threshold. The quality score from each base is calculated following the Phred+33 quality score encoding [45]. A minimum quality of 25 was used in the experiments.
- NONIUPAC: a single filter that removes sequences if they contain Non-IUPAC bases (that is, any base other than A, T, G, C or N).
- TRIMRIGHTP: a trimmer that trims sequences according to an indicated percentage of the total number of bases starting from the right. A 10% trimming was used in the experiments.
- DNATORNA: a formatter that transforms DNA sequences to RNA sequences.

In addition, two different scenarios have been tested depending on the state of the input dataset:

- “Downloaded”, where the full dataset is already locally stored in the master node (i.e., it was previously downloaded). In this scenario, SeQual first requires to copy the dataset to HDFS for processing it, since batch data processing can only begin once the complete dataset was copied. However, SeQual-Stream is able to read it directly from the local file system of the master node and start its processing while it is being copied to HDFS.
- “Downloading”, where the dataset is stored on a remote location outside the cluster (e.g., an external repository or server), so it must be first downloaded locally on the master node and then copied to HDFS for its processing. This second scenario serves to exploit one of the main advantages of our approach, as SeQual-Stream can start data processing as soon as the download operation is initiated, instead of waiting for the download to finish and copy the full dataset to HDFS. For simplicity, the datasets are stored on private servers instead of on a public repository to prevent that highly variable Internet download speeds could affect the results of these experiments. Each testbed has access to a separate private server with different download speeds: the first testbed downloads the datasets from a server providing speeds of 90–100 MiB/s, while the second one downloads them from a server with speeds of 600–700 MiB/s.

Finally, all the experiments were run a minimum of 5 times using the cluster nodes in a dedicated manner (i.e., the hardware was never shared by other users’ jobs running on the same cluster). Due to this fact, the observed standard deviations were not significant, and so the median value will be used as the performance metric in this work. Consequently, all the results shown in the next sections represent the median value of 5 measurements for each experiment.

**Table 1:** Hardware and software characteristics of the cluster nodes (testbed 1)

*Hardware*		
CPU Model	2 × Intel Xeon E5-2660 Sandy Bridge-EP	
CPU Speed/Turbo	2.20 GHz/3.0 GHz	
#Cores per node	16	
#Threads per node	32	
Cache L1/L2/L3	32 KiB/256 KiB/20 MiB	
Memory	64 GiB DDR3 1600 MHz	
Disk	1 × HDD 1 TiB SATA3 7.2K rpm	
Network	Gigabit Ethernet	
*Software*		
OS Version	CentOS Linux release 7.9.2009	
Kernel	3.10.0-1160.62.1	
Java	OpenJDK 1.8.0_322	
Spark	Version	3.1.1
	Executors per node	1
	Executor heap size	55 GiB
	Executor cores	16
Hadoop	Version	2.10.1
HDFS	Block size	128 MiB
	Replication factor	3

**Table 2:** Hardware and software characteristics of the cluster nodes (testbed 2)

*Hardware*		
CPU Model	2 × Intel Xeon Silver 4216 Cascade Lake-SP	
CPU Speed/Turbo	2.1 GHz/3.2 GHz	
#Cores per node	32	
#Threads per node	64	
Cache L1/L2/L3	32 KiB/1 MiB/22 MiB	
Memory	256 GiB DDR4 2933 MHhz	
Disks	1 × HDD 2 TiB SATA3 7.2K rpm	
	1 × SSD 240 GiB SATA3	
Network	InfiniBand FDR	
*Software*		
OS Version	CentOS Linux release 7.9.2009	
Kernel	5.4.233-1	
Java	OpenJDK 1.8.0_372	
Spark	Version	3.1.1
	Executors per node	1
	Executor heap size	225 GiB
	Executor cores	32
Hadoop	Version	2.10.1
HDFS	Block size	128 MiB
	Replication factor	3

**Table 3:** Characteristics of the public datasets used in the performance evaluation

Dataset	SRR567455	SRR11442499	SRR5893671
Tag	SRR56	SRR114	SRR589
Organism	Homo sapiens	Homo sapiens	Triticum aestivum
#Reads	2 $\times$ 251.9 M	2 $\times$ 250.3 M	2 $\times$ 359.5 M
Read length	76 bp	99 bp	160 bp
Size	2 $\times$ 45 GiB	2 $\times$ 62 GiB	2 $\times$ 120 GiB

### Experiments on testbed 1

This first group of experiments is performed on the first testbed (see Table 1). The “downloaded” scenario is evaluated using the first two datasets (SRR56 and SRR114, see Table 3) in both single- and paired-end mode, while the second scenario (“downloading”) is only tested with the second dataset for brevity of results, in single- and paired-end modes as well. The largest dataset (SRR589) is excluded from these experiments as it is so large and computationally expensive to process for this hardware that runtimes exceeded the time limits imposed on this testbed.

#### “Downloaded” scenario

Tables 4 and 5 show the execution times of both tools for the first scenario. It is important to note that the results for SeQual take into account the time required to copy the input dataset before starting its processing. The results shown in the tables are organized according to the quality control operation being performed, the dataset and its corresponding single- or paired-end layout, and the number of worker nodes used in the experiment (from 1 to 16). The last column shows the speedup obtained by our solution over its batch counterpart.

On the one hand, it can be observed that the maximum speedup achieved by our tool is 2.70$\times$, which is obtained for the QUALITY filter when processing the SRR56 dataset in paired-end mode. On the other hand, the average speedup for all operations and datasets under evaluation in this first scenario is around 1.43$\times$. In general, the speedups are usually higher when using a small number of worker nodes (1–4), whereas it tends to converge to 1 when using 16 nodes. The main reason for this behavior is that there comes a point where there is so much computational power and disks on which to spread the write operations that the processing and writing of the results are fast enough, whereas the speed of copying the input files, which is similar for both tools, becomes the major performance limiting factor due to the slow HDD disks available on this testbed.

There are also some differences to be pointed out between the results obtained for the different operations. The QUALITY filter and the TRIMRIGHTP trimmer tend to generate smaller runtimes for both tools and obtain greater speedups than the NONIUPAC filter and the DNATORNA formatter. This is due to the different amount of data written to HDFS as output for each operation. Whereas QUALITY removes sequences from the input dataset and TRIMRIGHTP makes them smaller, NONIUPAC does not filter any (because all bases are in IUPAC nomenclature in these datasets) and DNATORNA simply changes their format (thus maintaining all the sequences and their length). Consequently, these last two operations write more output data and impose a greater overhead on the disks, a more limiting factor in SeQual-Stream because the copy of the input dataset(s) is being made in parallel with the processing and writing of the results.

Overall, the speedups obtained tend to increase noticeably for paired-end experiments compared to single-end ones. In fact, all speedups greater than 2$\times$ are achieved in paired-end mode. It is important to remark that this mode involves copying and processing twice as much data as in single-end mode. As the amount of input data increases significantly, the time required to copy them to HDFS, process them with Spark and write their parts to HDFS also increases proportionally. Therefore, parallelizing all this process through a stream model is very beneficial and is precisely what was sought after with the development of this tool.

**Table 4:** Runtimes (in seconds) and corresponding speedups of SeQual-Stream over SeQual for different single- and paired-end datasets using the QUALITY and NONIUPAC filters (“Downloaded” scenario, testbed 1)

Operation	Dataset	Mode	Nodes	SeQual	SeQual-Stream	Speedup
QUALITY	SRR56	Single	1	1386	927	1.49
			2	993	708	1.40
			4	780	595	1.31
			8	566	507	1.12
			16	522	513	1.02
		Paired	1	5312	1967	2.70
			2	3639	1470	2.47
			4	2043	1102	1.85
			8	1119	949	1.18
			16	1024	940	1.09
	SRR114	Single	1	2239	1918	1.17
			2	1569	1070	1.47
			4	1242	941	1.32
			8	865	702	1.23
			16	723	689	1.05
		Paired	1	6983	4082	1.71
			2	5367	2278	2.36
			4	3126	1688	1.85
			8	2085	1368	1.52
			16	1452	1306	1.11
NONIUPAC	SRR56	Single	1	1430	1226	1.17
			2	1127	1081	1.04
			4	1013	703	1.44
			8	586	513	1.14
			16	539	518	1.04
		Paired	1	5919	2748	2.15
			2	4086	1958	2.09
			4	2328	1413	1.65
			8	1240	990	1.25
			16	1056	960	1.10
	SRR114	Single	1	2140	1896	1.13
			2	1600	1454	1.10
			4	1047	995	1.05
			8	815	682	1.19
			16	730	686	1.06
		Paired	1	6858	4549	1.51
			2	6044	3000	2.01
			4	3098	2079	1.49
			8	1646	1370	1.20
			16	1439	1328	1.08

**Table 5:** Runtimes (in seconds) and corresponding speedups of SeQual-Stream over SeQual for different single- and paired-end datasets using the TRIMRIGHTP trimmer and DNATORNA formatter (“Downloaded” scenario, testbed 1)

Operation	Dataset	Mode	Nodes	SeQual	SeQual-Stream	Speedup
TRIMRIGHTP	SRR56	Single	1	1682	1062	1.58
			2	934	872	1.07
			4	765	680	1.13
			8	583	523	1.11
			16	521	523	1.00
		Paired	1	5850	2259	2.59
			2	3400	1906	1.78
			4	2280	1463	1.56
			8	1354	961	1.41
			16	1051	961	1.09
	SRR114	Single	1	2855	1723	1.66
			2	1381	1281	1.08
			4	1050	909	1.16
			8	849	723	1.17
			16	729	688	1.06
		Paired	1	5960	4119	1.45
			2	5514	2391	2.31
			4	3329	1969	1.69
			8	1859	1390	1.34
			16	1420	1325	1.07
DNATORNA	SRR56	Single	1	1840	1588	1.16
			2	1194	1121	1.06
			4	918	673	1.36
			8	650	521	1.25
			16	543	509	1.07
		Paired	1	5072	3535	1.43
			2	4069	1942	2.09
			4	2556	1541	1.66
			8	1449	1062	1.36
			16	1057	950	1.11
	SRR114	Single	1	3149	1999	1.57
			2	2012	1116	1.80
			4	1146	1013	1.13
			8	859	683	1.26
			16	722	671	1.08
		Paired	1	6322	4923	1.28
			2	5547	2760	2.01
			4	3650	1954	1.87
			8	2288	1333	1.72
			16	1456	1286	1.13

#### “Downloading” scenario

Table 6 shows the execution times of both tools for the second scenario, where the dataset is downloaded from an external server with a bandwidth of up to 100 MiB/s, as mentioned earlier. Note that the results for SeQual take into account the time required to download and copy the full dataset before starting its processing. The results shown in the table follow the same format as in the previous scenario.

In this case, the speedups of our tool range from a minimum of 1.26$\times$ up to a maximum of 2.45$\times$, obtained for the QUALITY filter in single-end mode using 16 nodes and the DNATORNA formatter in paired-end mode using 4 nodes, respectively. The average speedup is around 1.71$\times$, which is a 20% higher than in the “Downloaded” scenario. Overall, the speedups are much better even when using 16 worker nodes, especially for the paired-end experiments. For instance, SeQual-Stream reduces SeQual execution times by 24% and 30% when applying TRIMRIGHTP over the SRR114 dataset using 16 nodes in single- and paired-end mode, respectively.

It is also worth noting how the speedup difference between single- and paired-end mode is slightly attenuated in this second scenario compared to the previous one. For instance, even though almost all speedups greater than 2$\times$ are also achieved in paired-end mode as before, there is at least one experiment where a 2.10$\times$ speedup is obtained in single-end (TRIMRIGHTP), and yet, the maximum speedup (2.45$\times$) is lower than in the first scenario (2.70$\times$). The main reason is that reading two input files requires copying both files simultaneously to join each sequence with its corresponding pair. When both files are being downloaded, it is very likely that one file is downloaded faster than the other, and so the additional data available from the faster file cannot be used by SeQual-Stream, as it is required to wait for the paired sequences to arrive from the slower file.

**Table 6:** Runtimes (in seconds) and corresponding speedups of SeQual-Stream over SeQual for the SRR114 dataset and different quality control operations (“Downloading” scenario, testbed 1)

Operation	Dataset	Mode	Nodes	SeQual	SeQual-Stream	Speedup
QUALITY	SRR114	Single	1	2599	1373	1.89
			2	1929	1134	1.70
			4	1602	1024	1.56
			8	1225	864	1.42
			16	1083	858	1.26
		Paired	1	7703	3490	2.21
			2	6087	2869	2.12
			4	3846	1920	2.00
			8	2805	1548	1.81
			16	2172	1518	1.43
NONIUPAC	SRR114	Single	1	2500	1818	1.37
			2	1960	1273	1.54
			4	1407	918	1.53
			8	1175	843	1.39
			16	1090	829	1.31
		Paired	1	7578	3611	2.10
			2	6764	2959	2.29
			4	3818	1891	2.02
			8	2366	1561	1.52
			16	2159	1559	1.38
TRIMRIGHTP	SRR114	Single	1	3215	1529	2.10
			2	1741	1174	1.48
			4	1410	1019	1.38
			8	1209	838	1.44
			16	1089	829	1.31
		Paired	1	6680	3363	1.99
			2	5874	2659	2.21
			4	4049	1828	2.21
			8	2579	1522	1.69
			16	2140	1501	1.43
DNATORNA	SRR114	Single	1	3509	1953	1.80
			2	2372	1678	1.41
			4	1506	1079	1.40
			8	1219	863	1.41
			16	1082	813	1.33
		Paired	1	7042	4290	1.64
			2	6267	2668	2.35
			4	4370	1780	2.45
			8	3008	1559	1.93
			16	2176	1488	1.46

### Experiments on testbed 2

This set of experiments is performed on the second testbed (see Table 2). The “downloaded” and “downloading” scenarios are both evaluated using the same four quality control operations and processing the largest dataset (SRR589, see Table 3), in this case only in paired-end mode for brevity of results. These experiments require the use of the HDD disks available on the cluster nodes, as the SSD disks do not have enough space to store both the input and output data for this huge dataset.

On the one hand, Table 7 shows the execution times of both tools for the “downloaded” scenario using up to 8 worker nodes. Overall, it can be seen that the speedups are very similar to those obtained for the analogous scenario on the first testbed (see paired-end results in Tables 4 and 5). Although the maximum speedup is slightly lower (up to 2.38$\times$), the average values remain practically the same (from 1.63$\times$ to 1.62$\times$). In these experiments, the speedups tend to be slightly lower due to the significantly higher computational power of the hardware. Consequently, the speed of copying the input files to the slow HDD disks becomes the main performance bottleneck using just 8 nodes instead of 16 as in the first testbed.

On the other hand, Table 8 shows the execution times for the “downloading” scenario. Note that the runtimes for SeQual remain the same as in the previous “downloaded” scenario. This is due to the higher download speed of the external server from which the datasets are downloaded, which is now fast enough so that the limiting factor in the download/copy pipeline to HDFS is the copy phase. SeQual-Stream runtimes are also very similar, although they tend to be slightly higher, suggesting that the streaming approach was able to take slightly more advantage of the fact that the input data was already complete. Compared to the analogous scenario evaluated on the first testbed (see paired-end results in Table 6), the speedup results tend to be lower, with an average of 1.60$\times$. Due to the faster download speed, there are fewer opportunities for overlapping and therefore fewer advantages for stream processing, although the maximum speedup achieved in these experiments (2.66$\times$) is still higher than before (2.45$\times$).

Overall, these experiments on the second testbed demonstrate that our tool can still achieve lower runtimes than its batch counterpart when using newer and faster hardware. However, SeQual-Stream can take even more advantage of slow/commodity hardware, as the chance for overlapping the download and/or copy of the datasets to HDFS with their processing is critical in this case. This proves that cutting-edge hardware is not necessary to use our tool, as good results can be obtained using commodity hardware such as in the first testbed.

**Table 7:** Runtimes (in seconds) and corresponding speedups of SeQual-Stream over SeQual for the SRR589 paired-end dataset (“Downloaded” scenario, testbed 2)

Operation	Dataset	Mode	Nodes	SeQual	SeQual-Stream	Speedup
QUALITY	SRR589	Paired	1	9369	5137	1.82
			2	7666	4629	1.66
			4	5152	3059	1.68
			8	3418	2728	1.25
NONIUPAC	SRR589	Paired	1	13253	5558	2.38
			2	7703	4498	1.71
			4	4887	3307	1.48
			8	3172	2818	1.13
TRIMRIGHTP	SRR589	Paired	1	13343	5875	2.27
			2	7532	4172	1.81
			4	4813	3018	1.59
			8	3085	2718	1.14
DNATORNA	SRR589	Paired	1	10256	5668	1.81
			2	7346	5468	1.34
			4	5179	3098	1.67
			8	3196	2758	1.16

**Table 8:** Runtimes (in seconds) and corresponding speedups of SeQual-Stream over SeQual for the SRR589 paired-end dataset (“Downloading” scenario, testbed 2)

Operation	Dataset	Mode	Nodes	SeQual	SeQual-Stream	Speedup
QUALITY	SRR589	Paired	1	9369	4924	1.90
			2	7666	4023	1.91
			4	5152	3601	1.43
			8	3418	2797	1.22
NONIUPAC	SRR589	Paired	1	13253	5894	2.25
			2	7703	5281	1.46
			4	4887	3478	1.41
			8	3172	2870	1.11
TRIMRIGHTP	SRR589	Paired	1	13343	5022	2.66
			2	7532	4285	1.76
			4	4813	3483	1.38
			8	3085	2891	1.07
DNATORNA	SRR589	Paired	1	10256	5632	1.82
			2	7346	5133	1.43
			4	5179	3070	1.69
			8	3196	2864	1.12

### Analysis of the scalability

This section presents a final set of experiments aimed at improving and analyzing the scalability of our streaming tool. In order to demonstrate its scaling capabilities, these experiments are focused on overcoming the main bottlenecks that limited performance so far.

For this purpose, the “downloaded” scenario has been evaluated using the second testbed (see Table 2), which provides SSD disks, to speed up the writing of both the input datasets and the results to HDFS. In addition, the input files have been previously copied to the SSD disk of the master node for faster read times. In these experiments, the same four operations have been executed using the largest dataset that can be processed in this testbed (i.e., SRR114, as SRR589 is too large to be stored on the SSD disks). Experiments with only one worker node must also use the HDD disk of the worker, as the full input dataset and the generated output do not fit entirely on its SSD. For the sake of simplicity, results are shown for paired-end mode only.

Table 9 shows the results obtained. Overall, the speedups are significantly higher, reaching a maximum value of 9.89x when using 8 nodes. It is interesting to note the significant differences between the operations. The NONIUPAC filter and the TRIMRIGHTP trimmer achieve the greatest speedups, followed by the DNATORNA formatter with a maximum of 6.26$\times$. The QUALITY filter gives the worst results, with a maximum of 4.72$\times$. As an attempt was made to remove all potential sources of bottlenecks in these experiments, most of the runtime corresponds to pure processing time and not to copying and/or writing the results. Therefore, the differences in runtimes and speedups are mainly due to the differences in performance and computational efficiency of each quality control operation.

**Table 9:** Runtimes (in seconds) and corresponding speedups of SeQual-Stream for the SRR114 paired-end dataset (“Downloaded” scenario, testbed 2 with SSD disks)

Operation	Dataset	Mode	Nodes	SeQual-Stream	Speedup over 1 node
QUALITY	SRR114	Paired	1	1440	1.00
			2	625	2.30
			4	590	2.44
			8	305	4.72
NONIUPAC	SRR114	Paired	1	1661	1.00
			2	818	2.03
			4	412	4.03
			8	186	8.93
TRIMRIGHTP	SRR114	Paired	1	1681	1.00
			2	1073	1.57
			4	553	3.04
			8	170	9.89
DNATORNA	SRR114	Paired	1	1541	1.00
			2	683	2.26
			4	535	2.88
			8	246	6.26

## Conclusion

The large amount of genomic data generated by modern NGS technologies reinforces the need for bioinformatics tools capable of reducing the time required for processing them as much as possible. In this paper we have presented SeQual-Stream, a Big Data tool for quality control of raw NGS datasets which seeks to reduce data processing times through exploiting Apache Spark and its Structured Streaming API. This combination allows our tool to take full advantage of distributed-memory systems such as clusters and to further accelerate quality control by overlapping data processing with downloading and/or HDFS copy operations.

The performance evaluation, conducted on two cluster testbeds using three publicly available datasets, has experimentally demonstrated that our stream approach can be up to nearly three times faster than the counterpart tool based on batch processing. This makes SeQual-Stream a useful tool in those cases where multiple large experiments need to be carried out, since such a speedup on each experiment would result in a significant overall improvement. This is especially significant when using small-scale clusters, which is a common computing facility that most biologists and bioinformaticians have access to. In fact, our results have also shown that a maximum speedup of around 10x can be achieved when using eight nodes compared to just using a single node.

As future work, we would be interested in adapting to the stream paradigm other quality control operations that perform their processing considering the whole set of sequences, which makes them much more complex to implement in streaming mode. The possibility of exploring the use of other stream processing frameworks such as Apache Flink is also of great interest.

## Availability and requirements

Project name: SeQual-Stream

Project home page: https://github.com/UDC-GAC/SeQual-Stream

Operating system(s): Platform independent

Programming language: Java

Other requirements: JRE 1.8 or higher, Apache Spark 3.0 or higher, Apache Hadoop 2.10 or higher (needed for HDFS)

License: GNU GPLv3

Any restrictions to use by non-academics: None

### Supplementary Information

**Additional file 1:** PDF document containing a detailed user's guide for SeQual-Stream.

## Data Availability

The software, documentation and source code of SeQual-Stream are publicly available at the GitHub repository: https://github.com/UDC-GAC/SeQual-Stream. The real datasets analyzed during this study are also publicly available at the NCBI SRA repository (https://www.ncbi.nlm.nih.gov/sra) using the accession numbers: SRR567455, SRR11442499 and SRR5893671.

## Abbreviations

NGS: Next generation sequencing
HDFS: Hadoop distributed file system
RDD: Resilient distributed dataset
SRA: Sequence read archive
NCBI: National Center for Biotechnology Information

## References

[1] Phillips KA (2018). Assessing the value of next-generation sequencing technologies: an introduction. Value Health.

[2] Minoche A, Dohm J, Himmelbauer H (2011). Evaluation of genomic high-throughput sequencing data generated on Illumina HiSeq and genome analyzer systems. Genome Biol.

[3] Edgar RC, Flyvbjerg H (2015). Error filtering, pair assembly and error correction for next-generation sequencing reads. Bioinformatics.

[4] He B (2020). Assessing the impact of data preprocessing on analyzing Next Generation Sequencing data. Front Bioeng Biotechnol.

[5] Zaharia M (2016). Apache Spark: a unified engine for big data processing. Commun ACM.

[6] Shvachko K, Kuang H, Radia S, Chansler R. The Hadoop distributed file system. In: Proceedings of the IEEE 26th symposium on mass storage systems and technologies (MSST 2010), Incline Village, NV, USA, (2010); 1–10.

[7] The Apache Software Foundation: structured streaming programming guide. https://spark.apache.org/docs/3.1.1/structured-streaming-programming-guide.html.

[8] Dean J, Ghemawat S (2008). MapReduce: simplified data processing on large clusters. Commun ACM.

[9] Ghemawat S, Gobioff H, Leung S-T. The Google file system. In: Proceedings of the 19th ACM symposium on operating systems principles (SOSP’03), Bolton Landing, NY, USA, 2003, pp 29–43

[10] The Apache Software Foundation: Apache Hadoop. https://hadoop.apache.org.

[11] Lakshman A, Malik P (2010). Cassandra: a decentralized structured storage system. ACM SIGOPS Oper Syst Rev.

[12] Zaharia M, et al. Resilient Distributed Datasets: A fault-tolerant abstraction for in-memory cluster computing. In: Proceedings of the 9th USENIX symposium on networked systems design and implementation (NSDI’12), San Jose, CA, USA, 2012, pp 15–28

[13] The Apache Software Foundation: Spark SQL, DataFrames and Datasets Guide. https://spark.apache.org/docs/latest/sql-programming-guide.html.

[14] The Apache Software Foundation: Spark Streaming Programming Guide. https://spark.apache.org/docs/latest/streaming-programming-guide.html.

[15] Thein KMM (2014). Apache Kafka: next generation distributed messaging system. Int J Sci Eng Technol Res.

[16] Chung W-C, Ho J-M, Lin C-Y, Lee D-T. CloudEC: A MapReduce-based algorithm for correcting errors in next-generation sequencing big data. In: Proceedings of the 2017 IEEE international conference on big data (IEEE BigData 2017), Boston, MA, USA, (2017);2836–2842.

[17] Abuín JM, Pichel JC, Pena TF, Amigo J (2015). BigBWA: approaching the Burrows-Wheeler aligner to big data technologies. Bioinformatics.

[18] Gordon A, Hannon GJ. FASTX-Toolkit: FASTQ/A Short-reads Pre-processing Tools. http://hannonlab.cshl.edu/fastx_toolkit.

[19] Schmieder R, Edwards R (2011). Quality control and preprocessing of metagenomic datasets. Bioinformatics.

[20] Fukasawa Y, Ermini L, Wang H, Carty K, Cheung M-S (2020). LongQC: a quality control tool for third generation sequencing long read data. G3 Genes Genom Genet.

[21] Kumar G, Ertel A, Feldman G, Kupper J, Fortina P (2020). iSeqQC: a tool for expression-based quality control in RNA sequencing. BMC Bioinform.

[22] Zhou Q, Su X, Wang A, Xu J, Ning K (2013). QC-Chain: fast and holistic quality control method for next-generation sequencing data. PLOS ONE.

[23] Cantu VA, Sadural J, Edwards R (2019). PRINSEQ++, a multi-threaded tool for fast and efficient quality control and preprocessing of sequencing datasets. PeerJ Preprints.

[24] Andrews, S. FastQC: A Quality Control Tool for High Throughput Sequence Data. https://www.bioinformatics.babraham.ac.uk/projects/fastqc/.

[25] de Sena Brandine G, Smith AD (2019). Falco: high-speed FastQC emulation for quality control of sequencing data. F1000Research.

[26] Chen Y (2017). SOAPnuke: a MapReduce acceleration-supported software for integrated quality control and preprocessing of high-throughput sequencing data. GigaScience.

[27] Expósito RR, Galego-Torreiro R, González-Domínguez J (2020). SeQual: big data tool to perform quality control and data preprocessing of large NGS datasets. IEEE Access.

[28] Melsted P, Halldórsson BV (2014). KmerStream: streaming algorithms for k-mer abundance estimation. Bioinformatics.

[29] Mohamadi H, Khan H, Birol I (2017). ntCard: a streaming algorithm for cardinality estimation in genomics data. Bioinformatics.

[30] Behera S, Gayen S, Deogun JS, Vinodchandran NV. KmerEstimate: a streaming algorithm for estimating k-mer counts with optimal space usage. In: Proceedings of the 9th ACM international conference on bioinformatics, computational biology, and health informatics (ACM-BCB 2018), Washington, DC, USA, (2018);438–447.

[31] Irber LC, Brown CT. Efficient cardinality estimation for k-mers in large DNA sequencing data sets. bioRxiv, (2016);1–5.

[32] Rathee S, Kashyap A (2018). StreamAligner: a streaming based sequence aligner on Apache Spark. J Big Data.

[33] Mushtaq H, Ahmed N, Al-Ars Z. Streaming distributed DNA sequence alignment using Apache Spark. In: Proceedings of the 2017 IEEE 17th International conference on bioinformatics and bioengineering (BIBE 2017), Washington, DC, USA, (2017);188–193.

[34] Valdes C, Stebliankin V, Narasimhan G (2019). Large scale microbiome profiling in the cloud. Bioinformatics.

[35] Mushtaq H, Ahmed N, Al-Ars Z (2019). SparkGA2: production-quality memory-efficient Apache Spark based genome analysis framework. PLOS ONE.

[36] Mushtaq H, Al-Ars Z. Cluster-based Apache Spark implementation of the GATK DNA analysis pipeline. In: Proceedings of the 2015 IEEE International conference on bioinformatics and biomedicine (BIBM’15), Washington, DC, USA, (2015);1471–1477.

[37] Tian Y (2022). Transposon insertions regulate genome-wide allele-specific expression and underpin flower colour variations in apple (Malus spp.). Plant Biotechnol J.

[38] Abuín JM, Pichel JC, Pena TF, Amigo J (2016). SparkBWA: speeding up the alignment of high-throughput DNA sequencing data. PLOS ONE.

[39] Li X, Tang Y, Wang L, Chang Y, Wu J, Wang S (2022). QTL mapping and identification of genes associated with the resistance to *Acanthoscelides obtectus* in cultivated common bean using a high-density genetic linkage map. BMC Plant Biol.

[40] Zheng X, Bai J, Meixia Y, Liu Y, Jin Y, He X (2020). Bivariate genome-wide association study of the growth plasticity of staphylococcus aureus in coculture with escherichia coli. Appl Microbiol Biotechnol.

[41] National Center for Biotechnology Information: The Sequence Read Archive (SRA). https://www.ncbi.nlm.nih.gov/sra.

[42] Kodama Y, Shumway M, Leinonen R (2011). The sequence read archive: explosive growth of sequencing data. Nucleic Acids Res.

[43] National Center for Biotechnology Information: NCBI. https://www.ncbi.nlm.nih.gov/.

[44] Wheeler DL (2007). Database resources of the National Center for Biotechnology Information. Nucleic Acids Res.

[45] Shi H, Li W, Xu X. Learning the comparing and converting method of sequence Phred quality score. In: Proceedings of the 2016 6th International conference on management, education, information and control (MEICI 2016), Shenyang, China, (2016);260–263.

