# Supplementary material for: SeQual-Stream: approaching stream processing to quality control of NGS datasets
Source: BMC Bioinformatics. 2023 Oct 27;24:403. doi: 10.1186/s12859-023-05530-7 (PMC10612204; doi:10.1186/s12859-023-05530-7)
Supplement: Supplementary file 1 — Additional file 1: PDF document containing a detailed user's guide for SeQual-Stream. [file 12859_2023_5530_MOESM1_ESM.pdf]

SeQual-Stream user's guide

Supplementary Material

Óscar Castellanos-Rodríguez, Roberto R. Expósito, and Juan Touriño

1 Introduction

SeQual-Stream is a Big Data tool that allows performing quality control operations (e.g., filtering, trimming) on NGS datasets in a scalable way, currently supporting single-end and paired-end reads in FASTQ and FASTA formats. To do so, it relies on the Apache Spark framework [1] and the Hadoop Distributed File System (HDFS) [2], taking advantage of distributed-memory systems such as clusters. It easily allows switching between batch and streaming modes, so that the latter enables data processing as they are downloaded and/or copied to HDFS. SeQual-Stream implements this stream processing mode upon the Spark Structured Streaming API [3].

2 Prerequisites

SeQual-Stream is implemented in "pure" (100%) Java code in order to maximize cross-platform portability. This makes it possible to support both Windows and UNIX-like operating systems (e.g., GNU/Linux, macOS). In fact, it should run on any platform with a supported version of Java (x86_64 and ARM64).

The software prerequisites to run SeQual-Stream are the following:

– Apache Spark framework version 3.0 or higher and the `spark-submit` command available on the system PATH.
– Java Runtime Environment (JRE) version 1.8 or higher compatible with Spark and the JAVA_HOME environment variable pointing to the JRE installation (or the `java` command available on the system PATH).
– Apache Hadoop version 2.10 or higher, since SeQual-Stream uses HDFS to store and process the input datasets on a distributed manner.

3 Download

To obtain SeQual-Stream, you can clone its repository on your system by executing the following command:

```
git clone https://github.com/UDC-GAC/SeQual-Stream
```

4 Compilation

To be able to compile SeQual-Stream's source code, the prerequisites are the following:

– Java Development Kit (JDK) version 11 or higher.
– Apache Maven [4] version 3.0 or higher.
– Hadoop Sequence Parser (HSP) library [5] version 1.0.

The JAR file corresponding to the HSP library is included in the repository, so you need to add such JAR into your Maven local repository. You can use the following Maven command from within the SeQual-Stream root directory:

```
mvn org.apache.maven.plugins:maven-install-plugin:2.5.2:
    install-file -Dfile=SeQual-Model/dependency-jars/hadoop-
    sequence-parser-1.0.jar -DgroupId=es.udc.gac -DartifactId
    =hadoop-sequence-parser -Dversion=1.0
```

Now, to compile SeQual-Stream, you just need to execute the following Maven command from within the SeQual-Stream root directory:

```
mvn package -DskipTests
```

This will generate a folder called *target* inside the SeQual-CMD and SeQual-GUI modules of the project, containing each one the appropriate JAR file. How to use each module is explained in the next section. Note that the first time you execute the previous command, Maven will download all the plugins and related dependencies it needs to fulfill the command. From a clean installation of Maven, this can take quite a while. If you execute the command again, Maven will now have what it needs, so it will be able to execute the command much more quickly.

5 Execution

5.1 SeQual-CMD

SeQual-CMD allows the processing of NGS datasets from a console interface. To do so, you just need to use the `spark-submit` command provided by Spark to launch the appropriate JAR file (*sequal-cmd.jar*) located at the *SeQual-CMD/target* directory.

The general syntax to execute SeQual-Stream is the following:

```
spark-submit [SPARK_ARGS] SeQual-CMD/target/sequal-cmd.jar [
    SEQUAL-STREAM_ARGS]
```

`SPARK_ARGS` are arguments not interpreted by SeQual-Stream itself, but forwarded to the Spark runtime[1]. For instance, the `--master` option may be used to set the master URL of the cluster, which depends on the cluster manager and execution mode (e.g., local[*], spark://HOST:PORT, yarn).

To set the specific operations to be performed over the input datasets, together with their necessary parameters, a Java properties file is used as input argument (`-c` option). SeQual-Stream provides a template properties file at the *etc* directory (*ExecutionParameters.properties*), which includes all the possible operations and parameters. Additionally, SeQual-CMD provides the `-g` option to generate a new template properties file.

`SEQUAL-STREAM_ARGS` are the actual arguments interpreted by SeQual-Stream, the available ones being the following:

- `-i <InputFile>`: Specifies the input file from where sequences will be read.
- `-di <InputFile>`: Specifies the second input file from where paired sequences will be read, in case of processing paired-end datasets.
- `-o <OuputDirectory>`: Specifies the output directory where the resulting sequences will be written to.
- `-c <ConfigFile>`: Specifies the path to the properties file.
- `-smc <SparkMasterConf>`: Specifies the Spark master configuration (local[*] by default).
- `-lc <LoggerConfLevel>`: Specifies the logger configuration for Spark and other libraries (ERROR by default).
- `-g`: Generates a template properties file within the path specified with `-o`.
- `-f`: Filters sequences following the specified parameters.
- `-fo`: Formats sequences following the specified parameters.
- `-t`: Trims sequences following the specified parameters.
- `-s`: Computes the statistics before and after performing other operations on the sequences.
- `-sfo`: Generates a single output file named *input-file-name-results.format* within the output directory, along with a folder named *Parts* containing the output files for each partition.

5.1.1 Local mode execution

In order to execute SeQual-Stream locally you must set the `--master` option to `local[N]`. `N` represents the number of threads to be used, so, even though only one node will be used for data processing, there will be still some parallelization in multi-core systems. If `N` is set to `*`, Spark will use as many threads as available cores on the system.

As an example, the following command trims the sequences of the dataset *sample.fastq* as specified on the *ExecutionParameters.properties* file and writes the output in the *output* directory, using the Spark local mode and 4 threads:

[1] https://spark.apache.org/docs/latest/submitting-applications.html#launching-applications-with-spark-submit

```
spark-submit --master local[4] SeQual-CMD/target/sequal-cmd.
    jar -i sample.fastq -o output -c ExecutionParameters.
    properties -t
```

If the input dataset was stored in the local file system instead of HDFS, we would need to indicate the input parameter as `file://<dataset_path>`. This is only valid in the streaming mode. For instance, in the same scenario as before:

```
spark-submit --master local[4] SeQual-CMD/target/sequal-cmd.
    jar -i file://$HOME/datasets/sample.fastq -o output -c
    ExecutionParameters.properties -t
```

The following example shows the same scenario as the first one, but with a paired-end dataset instead. So, we are using two input files (*sample1.fastq* and *sample2.fastq*):

```
spark-submit --master local[4] SeQual-CMD/target/sequal-cmd.
    jar -i sample1.fastq -di sample2.fastq -o output -c
    ExecutionParameters.properties -t
```

5.1.2 Cluster mode execution

To execute SeQual-Stream on a cluster, the `--master` option must be specifically set according to the cluster manager used. Currently, Spark supports four cluster managers[2]: Standalone, Mesos, YARN and Kubernetes.

For instance, the `--master` option must be set to `spark://<host>:<port>` when using the Spark standalone cluster mode[3], where `host` is the IP address of the cluster master node (or hostname if it can be resolved to an IP address) and `port` the port configured to be used (7077 by default).

The following command trims the sequences of the dataset *sample.fastq* in the same way as the first example previously shown, but now it is launched on a Spark standalone cluster, where the master server IP is 23.195.26.187. It is using the client deploy mode, thus launching the process that runs the main program (or driver program) outside the cluster:

```
spark-submit --master spark://23.195.26.187:7077 --deploy-
    mode client SeQual-CMD/target/sequal-cmd.jar -i sample.
    fastq -o output -c ExecutionParameters.properties -t
```

When using YARN as cluster manager[4], the cluster location is defined based on the Hadoop configuration, so HADOOP_CONF_DIR or YARN_CONF_DIR variables must be properly configured.

[2] `https://spark.apache.org/docs/latest/cluster-overview.html`
[3] `https://spark.apache.org/docs/latest/spark-standalone.html`
[4] `https://spark.apache.org/docs/latest/running-on-yarn.html`

The previous example can be executed on a YARN cluster using the cluster deploy mode (thus launching the driver program inside the cluster) with the following command:

```
spark-submit --master yarn --deploy-mode cluster SeQual-CMD/
    target/sequal-cmd.jar -i sample.fastq -o output -c
    ExecutionParameters.properties -t
```

5.2 SeQual-GUI

SeQual-GUI allows using a graphical user interface rather than the console, thus greatly simplifying its usage to non-computer science experts. This GUI has been implemented upon the JavaFX library [6].

To execute SeQual-GUI, you must also rely on the `spark-submit` command to do so. Unlike SeQual-CMD, only the Spark arguments are needed, so you still have to set parameters such as the master URL (although you may use the interface's field "Spark Master Conf"). You must launch the appropriate JAR file (*sequal-gui.jar*) which is located at the *SeQual-GUI/target* directory following this syntax:

```
spark-submit [SPARK_ARGS] SeQual-GUI/target/sequal-gui.jar
```

The graphical interface of SeQual-Stream is shown in Figure S1. This interface is mainly composed of 6 different sections (numbered in the figure):

1. Configuration section. Allows the user to specify different parameters, like the input file, the output folder, the log level, the use of the stream processing mode...
2. Filters section. Allows the user to select which filters should be applied, as well as their corresponding parameters.
3. Trimmers section. Allows the user to select which trimmers should be applied, as well as their corresponding parameters.
4. Formatters section. Allows the user to select which formatters should be applied.
5. Statistics section. Allows the user to select which statistics should be computed.
6. Output section. A console-like window that shows to the user useful information about the status of the data processing.

In order to execute SeQual-GUI, you need a JRE 1.8 flavour with bundled JavaFX support. For simplicity, it is recommended to use Oracle JRE 1.8, which already includes the required JavaFX libraries. Otherwise, you must ensure that such libraries are available on your system or that you use a JRE 1.8 flavour with them (i.e., BellSoft, Azu Zulu). Note that most JRE 1.8 versions from OpenJDK do not provide JavaFX libraries, which are available to be installed separately on

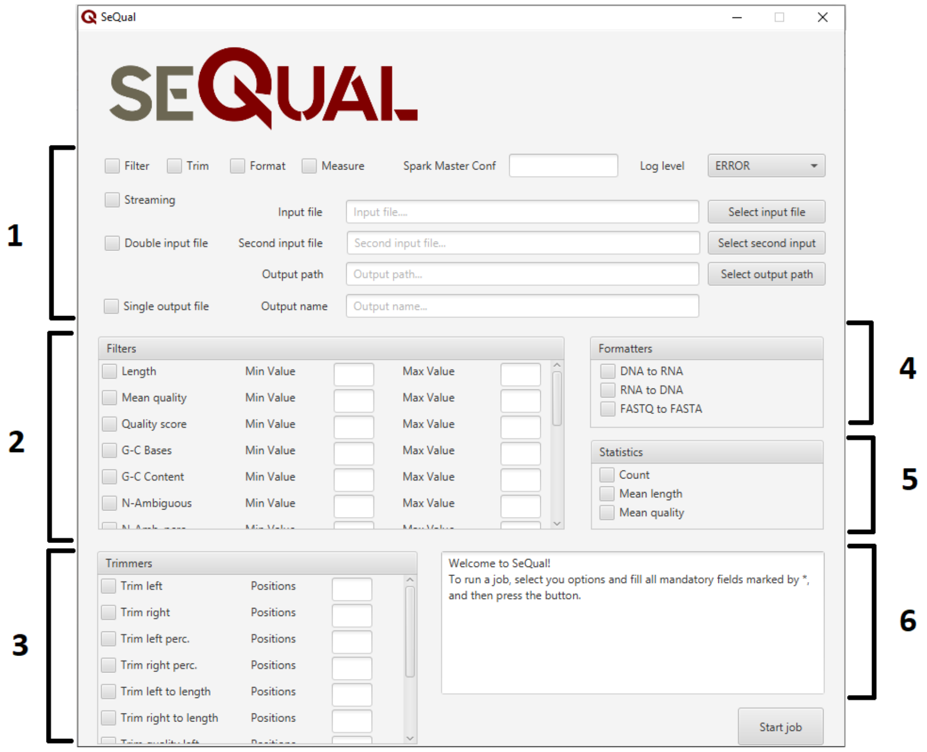

Figure S1. SeQual-Stream graphical interface

some systems. On UNIX-based systems, you can use the SDKMAN! manager [7] to install a JRE 1.8 flavour with JavaFX, which are those flavours listed with the FX suffix when executing the command `sdk list java`.

6 SeQual-Stream features

SeQual-Stream allows switching between batch or streaming processing modes using the `Mode` parameter within the properties file (or through the graphical interface when using SeQual-GUI). This file is also used to specify which operations are going to be performed, as well as their parameters, in SeQual-CMD. Note that SeQual-Stream command line arguments take priority; for instance, if a filter is selected on the properties file, it will not be executed unless the `-f` option is selected when running the `spark-submit` command. On batch processing mode four groups of features or operations that can be performed over the input datasets are offered, grouped based on the operation's objective. On streaming mode, a subset of these operations are supported. These groups are the following:

- Filters: They remove the sequences that do not comply with the specified thresholds specified by the user. There are single filters as well as group filters (only in batch mode).
- Trimmers: They trim the sequences following the specified parameters.
- Formatters: They apply data transformations to the sequences.
- Statistics (only in batch mode): They compute different statistics on the dataset.

Besides the already mentioned groups, there are other features grouped under the name Transversals, which allow the user to configure the application more thoroughly, specifying aspects like the log level or the Spark configuration. The SeQual-Stream features are detailed below:

- Single Filters:
  - `LENGTH`: Filters sequences based on an indicated maximum and/or minimum length threshold.
  - `QUALITYSCORE`: Filters sequences based on an indicated maximum and/or minimum quality score per base threshold, removing them if any of its bases is outside the threshold. Quality score from each base is calculated following Illumina encoding.
  - `QUALITY`: Filters sequences based on an indicated maximum and/or minimum mean quality threshold. Quality score from each base is calculated following Illumina encoding.
  - `GCBASES`: Filters sequences based on an indicated maximum and/or minimum quantity of G(uanine) and C(ytosine) bases threshold.
  - `GCCONTENT`: Filters sequences based on an indicated maximum and/or minimum GC-content threshold.
  - `NAMB`: Filters sequences based on an indicated maximum and/or minimum N-ambiguous bases quantity threshold.
  - `NAMBP`: Filters sequences based on an indicated maximum and/or minimum N-ambiguous bases percentage threshold.
  - `NONIUPAC`: Filters sequences if they contain Non-IUPAC bases (that is, any base other than A, T, G, C or N).
  - `PATTERN`: Filters sequences according to the absence of a specified pattern (that is, if it does not contain the pattern, the sequence is removed) along with its repetitions (for example, the pattern ATC with two repeats would be ATCATC).
  - `NOPATTERN`: Filters sequences according to the existence of a specified pattern (that is, if it does contain the pattern, the sequence is removed) along with its repetitions.
  - `BASEN`: Filters sequences according to whether they contain a maximum and/or minimum number of one or several base types (or even base groups).
  - `BASEP`: Filters sequences according to whether they contain a maximum and/or minimum percentage of one or several base types (or even base groups).

- Group Filters (only in batch mode):
  - `DISTINCT`: Filters duplicated sequences maintaining the ones with the highest quality (if they have associated quality).
  - `ALMOSTDISTINCT`: Filters duplicated sequences maintaining the ones with the highest quality (if they have associated quality), allowing an indicated margin of differences (for example, two sequences with two different bases can be considered equal if the specified limit allows it).
  - `REVERSEDISTINCT`: Filters reverse sequences maintaining the ones with the highest quality (if they have associated quality). For example, the reverse sequence of ATG is GTA.
  - `COMPLEMENTDISTINCT`: Filters complementary sequences maintaining the ones with the highest quality (if they have associated quality). For example, the complementary sequence of ATG is TAC.
  - `REVERSECOMPLEMENTDISTINCT`: Filters reverse complementary sequences maintaining the ones with the highest quality (if they have associated quality). For example, the reverse complementary sequence of ATG is CAT.
- Trimmers:
  - `TRIMLEFT`: Trims sequences according to an indicated number of positions starting from the 5'-end (left).
  - `TRIMRIGHT`: Trims sequences according to an indicated number of positions starting from the 3'-end (right).
  - `TRIMLEFTP`: Trims sequences according to an indicated percentage of the total number of bases starting from the 5'-end (left).
  - `TRIMRIGHTP`: Trims sequences according to an indicated percentage of the total number of bases starting from the 3'-end (right).
  - `TRIMQUALLEFT`: Trims sequences until achieving an indicated mean sequence quality starting from the 5'-end (left).
  - `TRIMQUALRIGHT`: Trims sequences until achieving an indicated mean sequence quality starting from the 3'-end (right).
  - `TRIMNLEFT`: Trims N-terminal tails with a specified minimum length at the 5'-end (left). An N-terminal tail is a set of N bases found at the beginning or end of a sequence. For example, the three Ns of the sequence NNNATCGAT form an N-terminal tail at the beginning.
  - `TRIMNRIGHT`: Trims N-terminal tails with a specified minimum length at the 3'-end (right).
  - `TRIMLEFTTOLENGTH`: Trims sequences to a specified maximum length starting from the 5'-end (left).
  - `TRIMRIGHTTOLENGTH`: Trims sequences to a specified maximum length starting from the 3'-end (right).
- Formatters:
  - `DNATORNA`: Transforms DNA sequences to RNA sequences.
  - `RNATODNA`: Transforms RNA sequences to DNA sequences.
  - `FASTQTOFASTA`: Transforms sequences in FASTQ format to FASTA format, losing the information of the quality.
- Statistics (only in batch mode):

  - `COUNT`: Calculates the total number of sequences in the dataset before and after performing the indicated operations on them.
  - `MEANLENGTH`: Calculates the mean length of the sequences in the dataset before and after performing the indicated operations on them.
  - `MEANQUALITY`: Calculates the mean quality of the sequences in the dataset before and after performing the indicated operations on them.
- Transversals:
  - Reading of FASTA format datasets: Allows to read datasets of sequences in FASTA format. In streaming mode, datasets can be stored in another file system besides HDFS and can be in the process of being downloaded.
  - Reading of FASTQ format datasets: Allows to read datasets of sequences in FASTQ format. In streaming mode, datasets can be stored in another file system besides HDFS and can be in the process of being downloaded.
  - Reading of paired-end FASTA format datasets: Allows to read datasets of paired-end sequences in FASTA format. The sequences must be separated in two different input files. In streaming mode, datasets can be stored in another file system besides HDFS and can be in the process of being downloaded.
  - Reading of paired-end FASTQ format datasets: Allows to read datasets of paired-end sequences in FASTQ format. The sequences must be separated in two different input files. In streaming mode, datasets can be stored in another file system besides HDFS and can be in the process of being downloaded.
  - Writing of resulting sequences: Allows to write the resulting sequences after the operations in the indicated path, generating two different folders in case of paired-end datasets. This type of writing is done by default, writing the result in several output text files. In streaming mode, the output text files are written into several subfolders.
  - Writing of resulting sequences to an individual file: Allows to write the resulting sequences after the operations to a single output file in the indicated path, or to two output files in case of paired-end datasets.
  - Configure Spark execution mode: Allows to configure the master URL for Spark, being local[*] by default (which implies using all the available cores in the machine where SeQual-Stream is executed).
  - Configure the level of log shown to the user: Allows to configure the log level shown to the user by Spark and other libraries. The default level is ERROR.
  - Generation and reading of a properties file: Allows to generate a template file where the operations to be carried out can be specified, as well as the necessary parameters for them.
  - Select between batch or streaming mode: Allows switching between batch or stream data processing modes.

References

1. Zaharia, M., *et al.*: Apache Spark: A unified engine for Big Data processing. Communications of the ACM **59**(11), 56–65 (2016)

2. Shvachko, K., Kuang, H., Radia, S., Chansler, R.: The Hadoop distributed file system. In: Proceedings of the IEEE 26th Symposium on Mass Storage Systems and Technologies (MSST 2010), Incline Village, NV, USA, pp. 1–10 (2010)
3. The Apache Software Foundation: Structured Streaming Programming Guide. https://spark.apache.org/docs/3.1.1/structured-streaming-programming-guide.html
4. The Apache Software Foundation: Apache Maven. https://maven.apache.org/
5. Expósito, R.R., González-Domínguez, J., Touriño, J.: HSRA: Hadoop-based spliced read aligner for RNA sequencing data. PLOS ONE **13**(7), 1–25 (2018). Article e0201483
6. Oracle Corporation: JavaFX. https://openjfx.io/
7. SDKMAN!: The Software Development Kit Manager. https://sdkman.io/
